# Relationship between somatosensory and visuo-perceptual impairments and motor functions in adults with hemiparetic cerebral palsy

**DOI:** 10.3389/fneur.2024.1425124

**Published:** 2024-07-17

**Authors:** Isabelle Poitras, Alexandre Campeau-Lecours, Catherine Mercier

**Affiliations:** ^1^Center for Interdisciplinary Research in Rehabilitation and Social Integration, CIUSSS de la Capitale-Nationale, Quebec City, QC, Canada; ^2^School of Rehabilitation Sciences, Laval University, Quebec City, QC, Canada; ^3^Department of Mechanical Engineering, Laval University, Quebec City, QC, Canada

**Keywords:** sensory function, motor skills, cerebral palsy, robotic exoskeleton, tactile function, visual function, bilateral coordination, position sense

## Abstract

**Introduction:**

Children with cerebral palsy (CP) exhibit a variety of sensory impairments that can interfere with motor performance, but how these impairments persist into adulthood needs further investigation. The objective of this study was to describe the sensory impairments in adults having CP and how they relate to motor impairments.

**Methods:**

Nineteen adults having CP performed a set of robotic and clinical assessments. These assessments were targeting different sensory functions and motor functions (bilateral and unilateral tasks). Frequency of each type of impairments was determined by comparing individual results to normative data. Association between the sensory and motor impairments was assessed with Spearman correlation coefficient.

**Results:**

Impairment in stereognosis was the most frequent, affecting 57.9% of participants. Although less frequently impaired (26.3%), tactile discrimination was associated with all the motor tasks (unilateral and bilateral, either robotic or clinical). Performance in robotic motor assessments was more frequently associated with sensory impairments than with clinical assessments. Finally, sensory impairments were not more closely associated with bilateral tasks than with unilateral tasks.

**Discussion:**

Somatosensory and visuo-perceptual impairments are frequent among adults with CP, with 84.2% showing impairments in at least one sensory function. These sensory impairments show a moderate association with motor impairments.

## Introduction

Cerebral palsy (CP) is the most common cause of physical disability through childhood (1). People living with hemiparetic CP display motor impairments predominantly on one side of the body and at one upper extremity, referred to as the more affected arm (MA), while the other upper extremity often displays milder deficits and is referred to as the less affected arm (LA). Recently, a scoping review from Brun et al. (2) has shown that most people living with CP also have somatosensory impairments in both of their arms, tactile perception and position sense being frequently impaired. Visual impairments are also highly prevalent among individuals with CP, with the majority (estimated prevalence ranging from 36 to 100%) exhibiting at least one visual abnormality, including refractive error, amblyopia, nystagmus or strabismus (3, 4). Additionally, 40 to 50% of children having CP experience visuo-perceptual impairments (5). Notably, most studies retrieved in the systematic review of (2) only or predominantly involved children, leaving a significant gap in the literature concerning adults. Recently, it has been shown that sensory impairments persist into adulthood, although some bothersome symptoms tend to decrease (6). However, another study reported a high frequency of impairments in the same population (7). A systematic review by (8) reported that the timing and location of lesions influence both motor and sensory impairments in children and young adults. However, only one study including middle-aged and older adults was included in the results (7). This highlights the need for more studies involving adults with CP, as it provides insight into how sensorimotor function evolves with the maturation of the central nervous system.

Executing precise movements and interacting effectively with environment requires the integration of information arising from various sensory modalities. Tactile information provides insights into an object’s texture, temperature, and shape, guiding decisions on aspects such as hand aperture and application of pressure on the object (9, 10). Proprioceptive information enables to judge limb movement (i.e., kinesthesia) and position (i.e., position sense) during a task (11, 12). Visual information gives inputs about the position and movement of objects in the environment (13, 14). An impairment in one or more sensory inputs, or in sensory processing and integration, can therefore lead to various motor impairments and functional limitations. In fact, while damages to the sensory (15, 16) and motor (17, 18) tracts have both been described in CP, a study conducted in children with CP reported a larger extent of damage to the thalamocortical tract compared to the corticospinal tract (19). This study also revealed a significant association between damage to the thalamocortical tract and motor impairments. A recent systematic review (20) identified a moderate association between clinical assessments of tactile perception (i.e., two-point discrimination and stereognosis) and motor functions. The literature focusing on the relationship between proprioceptive or visuo-perceptual impairments and motor functions was limited, but a trend for a moderate relationship was found. This review also highlighted some differences between the relationships found between sensory impairments and motor performance according to whether a bilateral or a unilateral task was used to assess motor performance. However, it was impossible to determine whether disparities observed between both types of tasks were due to different requirements between unilateral and bilateral motor control *per se*, or to the use of different types of assessment (unilateral motor functions being evaluated with objective assessments of a specific motor function while bilateral motor functions were often assessed based on self-report and targeting activities of daily living). Moreover, the impact of sensory impairments on motor functions could potentially differ depending on the arm assessed and the sensory functions observed. Indeed, it was demonstrated in healthy subjects that individuals tend to monitor more closely the movement of their dominant arm and rely more on proprioceptive feedback for their non-dominant arm (21), while the opposite was observed in adolescents with CP (22). This underscores the importance of evaluating the association between sensory and motor functions, and specifically addressing differences between unilateral and bilateral tasks and between arms.

A recent review by Kantak et al. (23) provides a structure to better understand the complexity of bilateral tasks. These authors characterized bilateral tasks based on two characteristics: (1) the symmetry of arm movements (asymmetric or symmetric task); and (2) the conceptualization of task goal (independent goals or common goal). The complexity of the task and of the movement production vary widely according to these task characteristics, highlighting a potential difference between performance in different types of motor task and sensory impairments.

The aim of this study was to describe the somatosensory and visuo-perceptual impairments in adults with CP and explore how they relate to motor impairments.

The first specific objective of this study was to compare the frequency of occurrence of various types of somatosensory and visuo-perceptual impairments in adults having CP with mild to moderate motor impairments, and to determine if they differ between the levels of impairment as characterized by the Manual Ability Classification System (MACS I to III). We hypothesized that adults with CP exhibit sensory impairments in both of their arms, with a higher frequency of impairments expected in the more affected (MA) arm compared to the less affected (LA) arm, and that all the assessments would allow to distinguish between the severity of impairments.

The second specific objective was to explore the relationship between the somatosensory or visuo-perceptual impairments and the impairments observed in bilateral (asymmetric independent goals and symmetric common goal tasks) and unilateral tasks. We hypothesized that the sensory and motor impairments would display a moderate association (*r*_s_ > 0.4).

## Methods

### Participants

Participants were enlisted through health records at the Centre intégré universitaire de santé et de services sociaux de la Capitale-Nationale (CIUSSS-CN), through patient organizations as well as through the mailing list of Université Laval. To be eligible, participants had to meet the following criteria: (1) being aged from 18 to 65 years old; (2) having a diagnosis of hemiparetic CP; (3) being able to perform a transfer with minor assistance (to sit in the robotic device); (4) having a level of I, II, or III (i.e., mild to moderate impairments) on the MACS. Exclusion criteria encompassed: (1) cognitive impairments or, (2) uncorrected visual problems interfering with the assessment tasks. The ethical approval for the study was granted by the local ethics committee (Ethics #2018-609, CIUSSS-CN), and all participants gave their written informed consent before participating.

The MACS level was the clinical assessment used to describe the ability of participants to handle objects during everyday life activities. A level of I represents mild, barely visible impairments, while a level of II represents a reduction in speed and accuracy of movements, and a level of III represents significant difficulties manipulating objects requiring task adaptation (24).

### Experimental setup

Participants were involved in two assessment sessions, lasting approximately 3 h each, and comprising several robotic tasks and clinical assessments. This study is a part of a larger project, and only results relevant to the research questions are presented in this article. For the other portions of the project, please refer to Poitras et al. (25–28). The first session comprised the robotic tasks [performed with a bilateral Kinarm Exoskeleton Lab (Kinarm, Kingston, Ontario)], and most clinical assessments. The second session included only the Observation-based assessment of the involvement of MA limb during functional tasks. The two sessions were less than 2 weeks apart, except in one participant (4-week gap due to being affected by Covid during this period). The robotic tasks comprised three motor tasks, a bilateral asymmetric independent goals task (Object Hit), a bilateral symmetric common goal task (Ball on Bar), and a unilateral task (Visually Guided Reaching), as well as a proprioceptive (position sense) task (Arm Position Matching). The clinical assessments also comprised three motor assessments involving either asymmetric independent goals bilateral tasks (i.e., Observation-based assessment of the involvement of MA limb), a symmetric common goal task (i.e., Two-Arm Coordination Test), and a unilateral task (i.e., Jebsen-Taylor Hand Function Test [JTHFT]) as well as three sensory tests, a tactile discrimination test (i.e., two-point discrimination test), a stereognosis (i.e., object recognition test) and a visuo-perceptual test (i.e., Motor-Free Visual Perception Test [MVPT]).

### Robotic assessment

**Figure 1:**
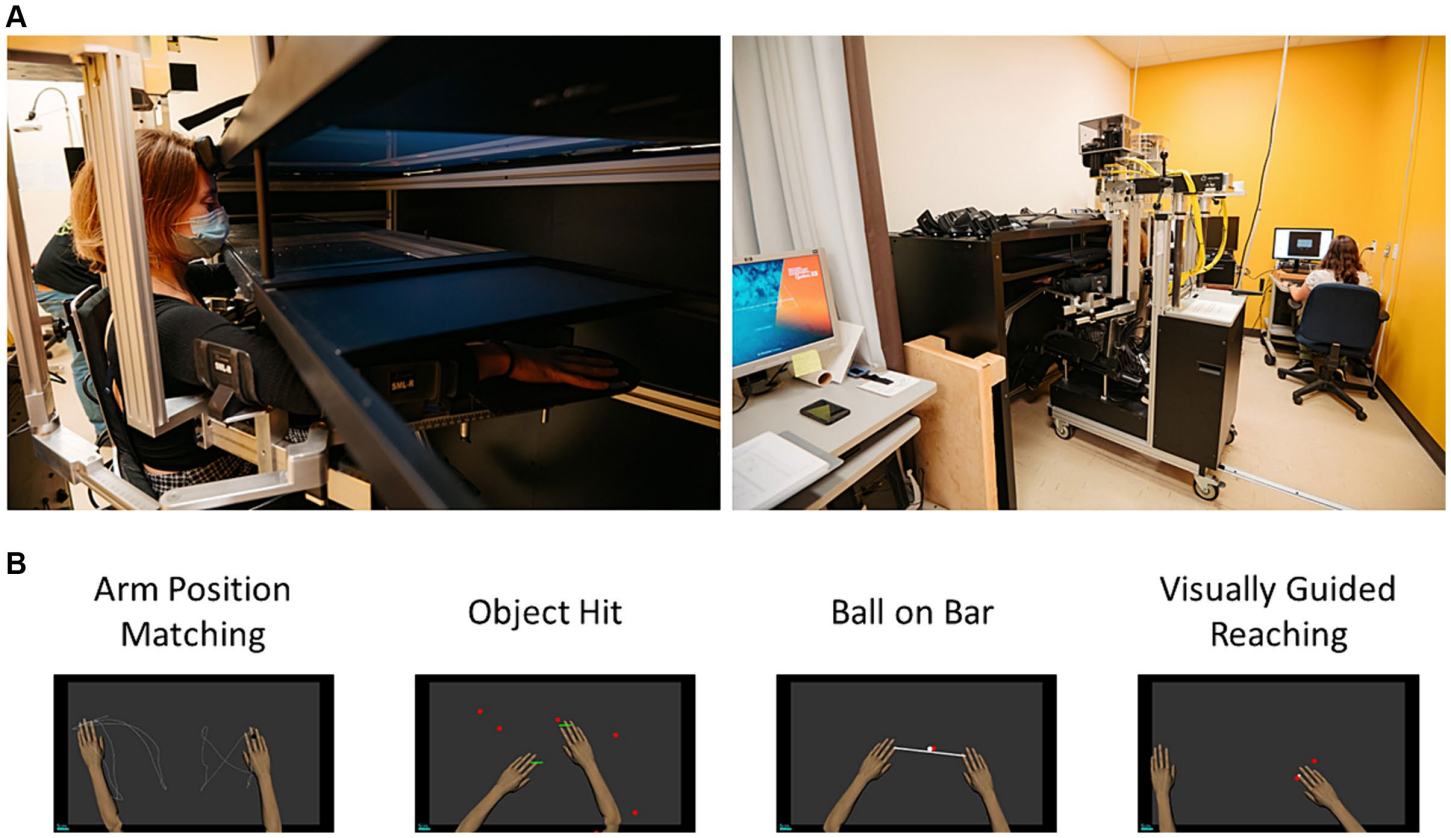
**(A)** Experimental setup for the Kinarm Exoskeleton Lab; and **(B)** Workspace representation of the four robotic tasks.

Figure 1 illustrates the experimental setup for the robotic assessment and provides an example of each task, that were executed in a predetermined sequence (matching the order of presentation outlined in the text), following the completion of anthropometric adjustments and calibration procedures. This robotic device has been used with different individuals having neurological disorder, including adults having a stroke (29), multiple sclerosis (30), Parkinson disease (31), and children having cerebral palsy (32) and developmental coordination disorder (33). The participant was positioned in the chair, with their arms placed within troughs integrated into a robotic arm system. This robotic setup enabled horizontal planar movements of the participant’s arm, encompassing flexion and extension actions at the shoulder and elbow joints, while reducing the influence of gravity. The robotic device did not assess fingers and wrists movements. Additionally, a 2D virtual-reality system was employed to manage visual stimuli and provide real-time feedback on the position of the arms. At the end of each experiment, the robotic device provided a csv file containing task-specific variables and a composite score called *Task-score*. The z-scores for task-specific variables were obtained by transforming the raw scores using a model constructed from data of healthy control (34). See Table 1 in the Supplementary materials for details on each task-specific variable. This transformation is detailed at (35). The *Task score* represents the assessment of the global performance during the task. This score was derived from a collection of task-specific variables, and the computational methodology was previously exposed in (34). To assess the comprehensive performance, the *Task score* was computed for each participant and each task (Object Hit, Ball on Bar, and Visually Guided Reaching, Arm Position Matching). Further information is available at (35). An individual with CP was considered to exhibit an impairment if their performance deviated beyond the 97.5% range observed in healthy controls (z-score < −1.96 or z-score > 1.96).

#### Somatosensory assessment

In the **Arm Position Matching task (Position sense)**, the robotic system displaced the MA arm in one position and the participant had to move their LA arm to mirror this position. The robotic device moved randomly the arm to one of the four positions (the four positions are 20 cm apart on a 2 × 2 grid) with a total of 24 trials by participants (36). The Arm Position Matching task was only executed using the MA arm moved by the robot, as the results could have been influenced by the limited motor ability to mirror the position accurately with the MA arm. It provides a quantitative measurement of proprioceptive impairments while allowing the identification of individuals with impaired proprioception by providing normative values matched for age, sex, and handedness. This assessment has been successfully used in children having CP (37–39) allowing to distinguish between healthy subject and neurologically impaired individuals, while psychometric properties of clinical assessments have been questioned and poorly reported (40).

#### Motor assessments

During the **Object Hit (asymmetric independent goals bilateral task)**, the participant was required to use both hands to strike balls that moved from the distant part of the screen to the closer part (i.e., toward them) across various medial-lateral positions (41). A total of 300 balls were presented, gradually increasing in speed. This task was repeated twice, and the analysis focused on the second trial to mitigate the impact of learning (42).

During the **Ball on bar (symmetric common goal bilateral task)**, a virtual bar was positioned between the participant’s hands, with a virtual ball placed on it, following the approach established by (43). The participant was presented with four targets successively, aiming to move the ball swiftly and accurately into each target. This task was divided into three levels, each lasting 1 min. In levels 2 and 3, the ball had the potential to “roll” and drop off the bar if tilted, necessitating precise bilateral control.

During the **Visually Guided Reaching (unilateral task)**, the participant’s objective was to point at four targets rapidly and precisely. These targets were distributed within a 10 cm radius from the initial target, and their presentation followed a pseudo-random sequence (resulting in a total of 24 reaching movements) (29, 32). This task was executed first with the less affected hand (LA arm) and subsequently with the more affected hand (MA arm).

### Clinical assessment

For the clinical assessment, the sensory assessments were performed first as they require more attention, followed by the Two-Arm Coordination Test and the JTHFT. All the assessments are described below.

#### Sensory assessment

During the **tactile discrimination assessment**, the participant’s hands were obstructed from view while the evaluator applied pressure longitudinally and perpendicularly to the tip of their index finger with a 2-Point Diskriminator (44). The aim of this assessment was to determine the minimal distance between two points at which a participant can discern the presence of two distinct points. The assessment started at 4 mm. If the participant successfully identified seven out of 10 trials at that distance, the gap between the two points was decreased; otherwise, it was increased. The participant was considered impaired if the minimal distance detected exceeded 6 mm (45). The LA arm assessment was always performed first, followed by the MA arm to make sure the participant fully understands the procedure.

The **stereognosis assessment (object recognition)** began with the presentation of 12 objects: a comb, a clothespin, a key, a spoon, a pen, a pencil, a diaper pin, a paperclip, a button, a penny, a marble and a small ball [based on (44)]. The participant had their hand obstructed from sight while the evaluator put one of the 12 objects in their hand. The objective of this assessment was to identify the object placed in their hand only by touching it. A set of the 12 objects were placed on the table in front of the participant allowing them to name or point the answer. The participant was characterized as impaired if they failed to identify one object. The LA arm assessment was always performed first, followed by the MA arm to make sure the participant fully understands the procedure.

The **Visuo-perceptual assessment (i.e., MVPT)** was developed to provide a general measure of visuo-perceptual processing capacities separated in five domains: visual discrimination, visual figure-ground, visual memory, visual closure and visual spatial unaffected by motor performance (46). The original version was used as it was quicker to administer the assessment while providing a good screening of their visuo-perceptual abilities. A participant was considered impaired if they fell outside the normative range for their age and sex as provided in the test scoring manual.

#### Motor assessments

An **Observation-based assessment of the involvement of MA limb (asymmetric independent goals bilateral task)** was used to quantify the degree of involvement of the MA limb in activities of daily living. The participant was asked to do seven simulated activities of daily living (1- cleaning up the table, 2- Making coffee, 3- Setting the table, 4- Pouring a glass of water, 5- Cutting a pied of mastic; 6- Folding towels; 7- Putting toothpaste on a toothbrush) while being video recorded. The rating was performed after the session by two evaluators. Observation-based assessment of the involvement of MA limb has been validated in (47). The score is the sum of 20 criteria (scale of 0 to 4) out of a total of 80.

The **Two-Arm Coordination Test (symmetric common goal bilateral task)** is an assessment of bimanual coordination during a constraint task. The apparatus consists in a star tracing board attached to two handles allowing moving a stylus on the drawing star (48). Pushing or pulling on the handles allows the stylus to move up and down and lateral displacements allows moving right and left. The participants had to perform four clockwise trials and four counterclockwise trials (randomly presented) with a 1 min of practice at the beginning of the testing. The measured variable is the mean Performance Index representing the time multiplied by the number of errors +1. The average of the third and the fourth trials was used for analysis as the performance is known to plateau after three trials (49). Since a Wilcoxon Rank test (*p* > 0.05) comparing clockwise and counterclockwise measures did not show a significant difference, the averaged Performance Index of both directions was employed.

The **JTHFT (unilateral task)** is a measure of fine and gross motor hand function using seven unilateral tasks: (1) writing a sentence; (2) turning cards; (3) picking up small objects; (4) simulating feeding; (5) stack checkers; (6) lifting, light objects, and (7) lifting heavy object. The MA hand is performed first and then the LA hand. The JTHFT is a standardized measure who was validated by assessing the MA arm first (50). The measured variable is the sum of the time needed to perform the seven tasks with each hand. The JTHFT have been validated (51).

### Statistical analysis

Descriptive statistics [including the mean, standard deviation (SD), and range] were computed for sociodemographic variables, as well as variables derived from robotic tasks and clinical assessments. All *Task score* derived from robotic assessments were transformed into z-scores using the normative data provided by the Kinarm company, which factored in sex, age, and laterality. The proportion of participants with a performance falling below the normal range was determined for each robotic task and each clinical assessment for which normative data were available (Objective 1). For all the statistical analyses, non-parametric tests were used given the limited sample size and the fact that some variables were not normally distributed (Performance Index of the Two Arm Coordination Test, JTHFT MA and LA arm, MVPT, TPD, stereognosis; Shapiro–Wilk tests with *p* < 0.05). Kruskal-Wallis tests were conducted to compare the results of the sensory assessments across different MACS levels (Objective 1). Spearman correlation coefficients were calculated between the results of the sensory assessments and the results of the motor assessments (Objective 2). The z-scores were used when normative data was available, while the raw score was used when no normative data was available (i.e., total score on 80 for the Observation-based assessment of the involvement of MA limb, and the performance index for the TACT). The correlations were categorized as follows: 0.00–0.09 as negligible; 0.10–0.39 as weak; 0.40–0.69 as moderate; 0.70–0.89 as strong; and 0.90–1.00 as very strong (52). Alpha threshold was set to 0.05. No correction for multiple comparisons was applied for correlation coefficients due to the exploratory nature of this objective and to the limited sample size.

## Results

### Sample description

Table 1 reports the demographic and clinical characteristics of the 19 participants, as well as their performance in the various motor assessments. One participant was unable to perform the Two Arm Coordination test due to a poor grasping capacity (i.e., unable to hold the handle of the device with the MA). All the participants were able to perform two attempts for the bilateral robotic tasks except one (S1) in which spasticity interfered with the completion of the second attempt. The data of the first trial was then kept for this participant. Note that although a learning effect in young athletes has been reported in the literature (42), a Wilcoxon signed-rank test showed no such effect in our sample (*p*-value>0.05). For a more detailed report on the results of the robotic motor tasks [see (35)].

**Table 1:** Demographic and clinical characteristics of the participants and their performance at the motor assessments (raw data or z-score).

Subject	Age	Sex	Affected side	Handedness*	MACS level	Object hit – Z score	Ball on bar – Z score	Visually guided reaching MA – Z score	Visually guided reaching LA – Z score	JTHFT MA – Z score	JTHFT LA – Z score	Observation-based assessment of the involvement of MA limb	Two-Arm coordination test
S1	28	F	R	L	1	0.59	1.36	**3.57**	**2.36**	**5.95**	**3.36**	70	45.9
S2	58	F	L	R	1	**3.96**	1.47	1.03	1.37	**7.14**	**6.31**	71	379.7
S3	49	F	R	R**	1	**2.61**	0.16	1.06	−0.56	1.29	**2.62**	80	40.06
S4	22	F	L	R	1	**2.15**	−0.53	0.73	1–07	**2.26**	0.47	77	43.4
S5	33	M	L	R	1	1.39	0.46	1.70	−0.66	**4.17**	0.46	71	98.9
S6	31	M	L	R	1	1.10	−0.51	−1.22	−0.66	1.84	**2.27**	80	55.1
S7	48	F	R	L	1	**3.72**	1.77	**3.44**	1.64	**2.61**	**2.24**	63	126.1
S8	53	F	R	L	2	1.58	**2.02**	**2.63**	1.26	**12.22**	**5.6**	55	341.9
S9	21	F	R	L	2	**2.42**	**2.69**	1.42	1.70	**7.50**	1.31	56	123.0
S10	31	F	R	L	2	**2.65**	**2.11**	**2.23**	0.86	**6.64**	**2.21**	58	96.2
S11	25	F	R	L	2	**4.18**	0.82	1.71	−0.13	**41.52**	**2.47**	51	59.8
S12	26	M	L	R	2	**2.40**	1.00	**3.35**	**2.16**	**13.70**	1.54	52	78.0
S13	41	F	L	R	2	**5.83**	**1.96**	**7.31**	1.71	**31.27**	**10.27**	59	313.8
S14	48	M	R	L	3	**2.48**	**3.82**	**5.02**	**3.32**	**6.98**	−0.19	61	47.8
S15	30	M	L	R	3	**3.70**	1.90	**2.08**	1.85	**74.98**	**5.95**	36	447.7
S16	26	M	R	L	3	**4.48**	**3.70**	**5.57**	**3.25**	**3.04**	1.15	71	98.1
S17	35	M	R	L	3	**3.08**	1.53	**2.51**	−0.34	**128.451**	1.12	26	161.02
S18	25	F	R	L	3	**4.56**	**4.09**	**5.89**	**5.07**	**121.69**	**33.31**	41	**-**
S19	23	M	R	L	3	**5.37**	**2.50**	**5.31**	**2.50**	**19.36**	**6.39**	48	242.04
**% impaired**						**78.9%**	**42.1%**	**63.1%**	**31.6%**	**89.4%**	**63.2%**		

Results are presented in bold when they fall below the normative data. MACS, Manual Ability Classification Scale; F, Female; M, Male; MA, More affected; LA, Less affected; R, Right; L, Left; JTHFT, Jebsen-Taylor Hand Function Test; * self -reported handedness, ** the only participant using their more affected arm as their dominant arm, − one participant was unable to perform the Two-Arm Coordination test.

### Objective 1: description of sensory impairments

**Figure 2:**
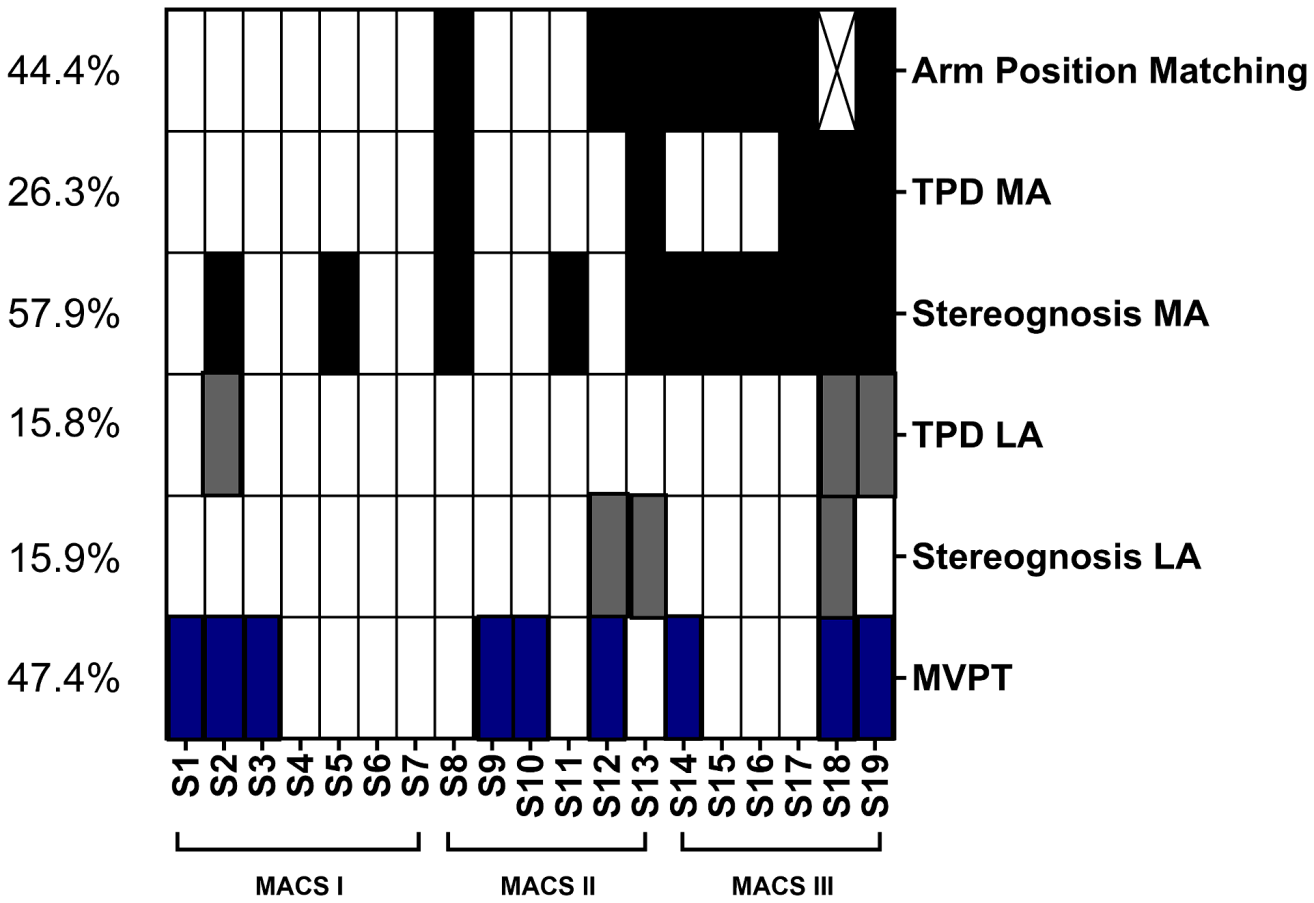
Distribution of participants with impairments in the different sensory assessments across the Manual Ability Classification Scale (MACS). The percentage of participants with an impairment in each function appears in the left column. The black boxes represent impairments on the more affected (MA) arm, gray boxes represent impairments in the less affected (LA) arm and the blue boxes the non-lateralized visuo-perceptual impairments (Motor-Free Visual Perception Test [MVPT]). The X represents a missing value for the participant that was unable to perform the Arm Position Matching task due to spasticity. TPD, Two-point Discrimination; MA, more affected; LA, less affected.

Figure 2 represents the distribution of sensory impairments across participants. The assessments reporting the highest rate of impaired participants were the stereognosis of the MA arm (57.9%), the Arm Position Matching (44.4%) and the visuo-perceptual test (47.4%). One participant was unable to perform the Arm Position Matching due to an increase in spasticity during this test involving passive arm displacement (S18). Although some participants exhibited deficits on the LA arm on the two-point discrimination and stereognosis tests, the occurrence of impairments on the MA arm was higher. The position sense test (*p* = 0.003), the tactile discrimination test of the MA arm (*p* = 0.02) and the stereognosis test of the MA arm (*p* = 0.014) allowed to distinguish across MACS levels. However, the participants with impairments in the visuo-perceptual test were evenly distributed across MACS levels, with three participants displaying impairments in each level. Noteworthy, three participants displayed no sensory impairments, and all of them had a MACS level I. Also, no participant having a MACS level of I exhibited proprioception impairments, and no participants with a MACS level of II displayed tactile discrimination impairments in their LA arm.

### Objective 2: relationship between the sensory impairments and motor impairments in bilateral and unilateral tasks

Table 2 reports the Spearman correlation coefficients between the sensory and the motor assessments. For the Arm Position Matching, a decrease in proprioception capacity (represented by a higher z-score) was associated with a decrease in motor performance during the Ball on Bar, the Visually Guided Reaching of the MA and LA arms, the Observation-based assessment of the involvement of the MA arm, and the JTHFT. For the tactile discrimination of the MA arm, a diminished capacity in tactile perception (represented by a larger distance between the two points detected) was associated with a decrease in motor performance in all tasks. Tactile discrimination and stereognosis of the LA arm exhibited no association with motor tests. For the stereognosis of the MA arm, a decrease in object recognition capacities (represented by a lower number of objects recognized) was associated with a decrease in motor performance during the Object Hit, the Ball on Bar, the Visually Guided Reaching of the MA arm, and the JTHFT of the MA arm. For visual perception, a decrease in visual perception capacities (represented by a decrease in the MVPT score) was associated with a decrease in motor performance of the Visually Guided Reaching of the LA arm.

**Table 2:** Spearman correlation coefficient between the somatosensory and visuo-perceptual assessments and the motor assessments (bilateral and unilateral).

	OH	BOB	VGR MA arm	VGR LA arm	Observation-based assessment of the involvement of MA arm	TACT	JTHFT MA arm	JTHFT LA arm
Arm position matching	0.39	0.67**	0.74***	0.62**	−0.74***	0.42	0.69**	0.08
Two-point discrimination test (MA arm)	0.53*	0.51*	0.56*	–	−0.57*	0.65**	0.66**	–
Two-point discrimination test (LA arm)	0.42	0.29	–	0.20	−0.42	0.21	–	0.31
Stereognosis (MA arm)	−0.50*	−0.46*	−0.52*	–	0.39	−0.43	−0.55*	–
Stereognosis (LA arm)	−0.33	−0.21	–	−0.41	0.30	−0.10	–	−0.39
MVPT	0.06	−0.44	−0.33	−0.53*	0.21	−0.01	−0.28	−0.24

MA, More Affected; LA, less affected; OH, Object Hit; BOB, Ball on Bar; VGR: Visually Guided Reaching; MVPT: Motor-Free Visual Perception Test; TACT: Two-Arm Coordination Test; JTHFT, Jebsen-Taylor Hand Function Test. *p < 0.05, **p < 0.01, ***p < 0.001. Significant results are in bold. Robotic assessments are represented by a light gray box and clinical assessment by a white box.

When comparing the different sensory tests, the tactile discrimination of the MA arm was the sensory assessment that was the most frequently associated with the motor assessments (six motor assessments out of six). The position sense test and the stereognosis of the MA arm were also frequently associated with the motor assessments (respectively five out of eight and four associations out of six tests). The visuo-perceptual test was only associated with the Visually Guided Reaching of the LA arm (robotic assessment).

When comparing robotic and clinical assessments, robotic assessments were the most frequently associated with the sensory impairments (10 significant associations vs. six). Interestingly, this difference is mainly due to the lower number of associations between the clinical assessments and the stereognosis test even if the clinical assessments require more fine distal movements (e.g., holding handles or writing) than the robotic assessments (requiring only shoulder and elbow movements). When comparing bilateral and unilateral tasks, the number of associations for each type of motor task with sensory impairments was similar for tactile discrimination and stereognosis (i.e., specifically all the assessments where associated with tactile discrimination, and there were two associations for both types of tasks for stereognosis), while Arm Position Matching was more frequently associated with unilateral tasks (i.e., 75% of the tests for the unilateral tasks, and 50% of them for the bilateral tasks). This difference is mainly due to the low number of associations found for the bilateral clinical tasks. Across the bilateral tasks, no difference was observed across asymmetric and the symmetric tasks between associations with sensory impairments.

Finally, the majority of relationships observed were moderate, except for two strong associations between the position sense test and the Visually Guided Reaching of the MA arm and the Observation-based assessment of the involvement of MA limb.

## Discussion

The aim of this study was to describe the somatosensory and visuo-perceptual impairments in adults living with CP and explore how they relate to motor impairments. Proprioception and stereognosis were the most frequently impaired somatosensory functions and allowed to discriminate between severity of manual impairments (MACS level). The absence of proprioceptive impairments for participants with a MACS level of I and the absence of tactile discrimination impairments in participants with a MACS level of II strengthen this finding. Although less frequently impaired, tactile discrimination of the MA arm was associated with motor impairments in all tasks. Visuo-perceptual impairments was also frequently impaired, but those impairments were evenly distributed across severity of manual impairment, and an association was found with a single motor test (Visually guided reaching od the LA arm). The motor impairments observed during bilateral and unilateral tasks were both frequently associated with somatosensory and visuo-perceptual impairments. A difference between the frequency of association with unilateral or bilateral tasks was observed only with the Arm Position Matching, while being similar for the tactile discrimination and the stereognosis. This result suggests that Arm Position Matching may have unique sensory processing or motor control demands that differentiate it from tactile discrimination and stereognosis, potentially indicating a greater sensitivity to the differences between unilateral and bilateral tasks.

Overall, the frequency of impairments in our group of adults with CP is slightly lower than the one described in children with CP for all somatosensory functions, but not for visuo-perceptual functions. Specifically, the frequency of tactile discrimination impairments in our group is 26.3%, as opposed to reported prevalences between 30 and 90% in children (53–55). Regarding stereognosis, the frequency of impairments in our group is 57.9%, compared to the literature-reported range of 77–97% (53, 54). In terms of proprioception, the observed frequency of impairments is 44.4% compared to the reported prevalence of 46 to 66% in children (2, 53, 54). For visuo-perceptual impairments, the frequency of impairments observed in our group is similar to the one reported in children [i.e., 47.4% compared to 40–50% in Ego et al. (5)]. These results indicate that a portion of sensory deficits persist over time despite central nervous system maturation. The lower frequency observed in adults might either be explained by such maturation (i.e., some deficits observed in children might represent a developmental delay), but also to the fact that assessment of somatosensory functions in children with CP is particularly challenging as they require a substantial level of attention. However, it is important to note that these observations are based on different protocols and a heterogeneous population, which only allows for a descriptive comparison between children and adults. A study involving children and adults with comparable clinical characteristics and a similar testing protocol would be needed to address this question directly.

Overall, the association between somatosensory and visuo-perceptual impairments and motor impairments was moderate, while being frequently observed. Potential explanations to this relationship can be separated in three different hypotheses. First, and the most often assumed hypothesis, is that precise and reliable sensory inputs are necessary to support motor coordination through feedback mechanisms. However, this hypothesis cannot account for the fact that robotic assessments that do not require manual functions were associated with tactile discrimination and stereognosis. An alternative hypothesis (although both are not mutually exclusive) is that precise and reliable sensory inputs plays a critical role in updating the feedforward model (56), and therefore sensory deficits would result in motor planning dysfunction. This becomes particularly relevant as individuals with CP commonly manifest diverse motor planning problems (57). Moreover, we recently demonstrated that feedforward deficits are more frequent than feedback deficits in adults with CP [Poitras et al. (35)]. According to these first two hypotheses, somatosensory or visuo-perceptual impairments would play a causal role in poorer motor functions. The third hypothesis is that the observed association is an epiphenomenon, simply reflecting the fact that individuals with larger brain lesions affecting corticospinal pathways are more likely to also have lesions affecting sensory pathways (19, 58). This can contribute to explain why less associations are observed between visuo-perceptual and motor functions, as pathways involved are anatomically more distinct. In future studies, it would be of interest to obtain brain imaging as well as an assessment of oculomotor behavior to investigate the relation between visuo-perceptual and motor functions in more details.

### Clinical implications

The results presented in this article could help understand the mechanisms underlying motor impairments in individuals with CP. This article confirms that sensory impairments persist into adulthood and are associated to some extent with motor impairments. Sensory impairments have historically been under-assessed and undertreated in patients; this article shows that this is an important concern that should be part of a comprehensive assessment in individuals with CP. Further studies could address interesting questions related to the hypotheses formulated above. For example, do sensory interventions indirectly improve motor function in adults and children with CP? If improvement occurs, is it linked to an improvement in motor planning, suggesting a better capacity to update the feedforward model, or to a better capacity to use online feedback for movement correction?

The results presented in our article should serve as a foundation to support further studies addressing the link between sensory impairments and motor control in adults with CP. Furthermore, findings in adults with CP could also help manage impairments in children. Indeed, if sensory impairments contribute significantly to motor impairments, managing sensory impairments at a young age should be prioritized.

### Study limitations

This study had some limitations that need to be acknowledged. Firstly, the number of participants was relatively small in a very heterogenous population, limiting the generalization of the results. However, the even distribution among MACS levels reduces potential bias. Secondly, the use of some clinical assessments without normative value of reference (i.e., Observation-based assessment of the involvement of MA limb and Two-Arm Coordination test) could explained the smaller frequency of associations observed with these assessments. However, no objective assessments of bilateral coordination with normative data were available in adults with CP, the other assessments available being self-reported assessments [e.g., ABILHAND (59)]. This highlights the need of more high-quality studies on adults having CP. Finally, the robotic system used is not representative of activities in the real-word, restricting the conclusions made regarding the implication of somatosensory and visuo-perceptual impairments during activities of daily living.

## Conclusion

The most frequently impaired sensory functions in adults with CP were stereognosis, joint position sense, and visuo-perceptual functions. The associations between somatosensory and visuo-perceptual functions observed were moderate for all the somatosensory and visuo-perceptual impairments. There is no difference observed between bilateral and unilateral tasks, suggesting that sensory impairments contribute to poorer motor in both types of tasks. Our results suggest that a comprehensive assessment of sensorimotor functions in adults living with CP should incorporate somatosensory and visuo-perceptual assessments.

## Data availability statement

The raw data supporting the conclusions of this article will be made available by the authors, without undue reservation.

## Ethics statement

The studies involving humans were approved by Centre intégré universitaire de santé et de services sociaux de la Capitale-Nationale, Ethics #2018-609. The studies were conducted in accordance with the local legislation and institutional requirements. The participants provided their written informed consent to participate in this study. Written informed consent was obtained from the individual(s) for the publication of any potentially identifiable images or data included in this article.

## Author contributions

IP: Writing – original draft, Methodology, Investigation, Formal analysis, Data curation, Conceptualization. ACL: Writing – review & editing, Supervision, Conceptualization. CM: Writing – review & editing, Supervision, Project administration, Investigation, Funding acquisition, Conceptualization.
