# Supplementary material for: Relationship between somatosensory and visuo-perceptual impairments and motor functions in adults with hemiparetic cerebral palsy
Source: Front Neurol. 2024 Jul 17;15:1425124. doi: 10.3389/fneur.2024.1425124 (PMC11290339; doi:10.3389/fneur.2024.1425124)
Supplement: Supplementary file 1 [file Table_1.docx]

Supplementary Material

Table 1: description of the task-specific variables calculated for each robotic assessment.

| Tasks | Task-specific variables | Definition |
| --- | --- | --- |
| Object Hit | Target hits | Percentage of balls hits by the participant during the whole task. The ball must move off the screen to be considered as hit. |
|  | Median error | Percentage of the task where the participant missed half the balls (errors). The result is based on the number of balls missed. |
|  | Miss bias | Distance in cm at which the participant missed the same number of balls on each side. |
|  | Hand speed (right and left) | Mean hand speed of each hand during the whole task. |
|  | Movement area (right and left) | Area of the workspace where the participant hit balls during the whole task. |
|  | Hand bias hits | A value from -1 to 1 representing which hand is used more often during the task. A value o -1 represent that the left hand hit more balls and a value of 1 that the right hand hit more balls. |
|  | Hand transition | A value in cm where the participant switches their hand preference to hit the balls. |
|  | Hand selection overlap | Percentage representing the efficiency of using both hands together (based on the number of times the hand switches to hit a ball, and on how often a ball is hit with both hand). |
|  | Hand speed bias | A value ranging from -1 to 1 representing whether one hand moves faster than the other. A value of -1 indicates that the left hand moves faster, while a value of 1 indicates that the right hand moves faster. |
|  | Movement area | A value ranging from -1 to 1 representing whether the hands move more to one side of the workspace than the other. A value of -1 indicates that the hands move more frequently on the left side of the workspace, while a value of 1 indicates that the hands move more frequently on the right side. |
| Ball on Bar | Targets complete | Number of targets successfully reached. |
|  | Drops | Number of times the ball fell of the bar. This task-specific variable is only calculated for the level 2 and 3, as the ball does not move in level 1. |
|  | Drops/Target | Ratio of the last two specific variables, Drops and Targets complete. |
|  | Ball speed | Mean speed of the ball by level. |
|  | Bar tilt stdev | Standard deviation of the bar tilt during the whole task. |
|  | Bar length variability | Coefficient of variation of the bar representing the standard deviation of bar length / mean bar length by level. |
|  | Hand speed difference | Difference between the hand speed of both hands. |
|  | Hand speed bias | Ratio of the number of hand speed peaks by hands representing whether one hand have more hand speed peaks than the other. |
| Visually Guided Reaching | No init stabilization | Number of trials where the participant is unable to stay still inside the initial target. |
|  | No end movement | Number of trials where no end of the reach was detected (e.g., the target was not reach, or the participant is unable to stay still in the target). |
|  | Posture speed | Median hand speed when the hand is supposed to be at rest. |
|  | Reaction time | Time between target appearance and the beginning of hand movement. |
|  | Initial direction angle | Angle between a line traced from the a) hand position at the beginning of the movement to the hand position after the initial phase, to b) the hand position at the end of the movement to the end target. The median across trial is calculated |
|  | Initial distance ratio | Ratio between the distance the hand travelled during the initial phase of the movement and the distance the hand travelled between the start and the end of the movement. The median across trial is calculated. |
|  | Speed maxima count | Mean number of maximum hand speed across trial. |
|  | Min-Max speed | Mean difference between two hand speed minimum and maximum after having reach the Maximum speed and before the end of the reach. The mean value across trial is calculated. |
|  | Movement time | Total time between the beginning and the end of the reach. |
|  | Path length ratio | Ratio between a) the distance travelled during the reach, and b) the shortest distance between the initial and final hand position. |
|  | Max speed | Maximum hand speed reach during a trial. The median value across trial is calculated. |
| Arm Position Matching | Absolute error X | Mean error in the X direction. |
|  | Absolute error Y | Mean error in the Y direction |
|  | Variability X | Mean value of the standard deviations of the hand in the X direction. |
|  | Variability Y | Mean value of the standard deviations of the hand in the Y direction. |
|  | Contraction/ Expansion ratio X | Ratio of the a) movement of the participant in the X direction, and b) movement performed by the robot in the X direction. |
|  | Contraction/ Expansion ratio Y | Ratio of the a) movement of the participant in the Y direction, and b) movement performed by the robot in the Y direction. |
|  | Shift X | Lateral or medial shift of the mirrored movement. Positive value represents a lateral shift, while a negative value represents a medial shift. |
|  | Shift Y | Distal or proximal shift of the mirrored movement. Positive value represents a distal shift, while a negative value represents a proximal shift. |

*Legends: Only the task-specific variables used to calculate the* Task score *are presented here. The descriptions are based on the one provided by the company (Kinarm 2024)*

Kinarm. 2024. "Kinarm Standard Tests." accessed June 13 2024. <https://kinarm.com/kinarm-products/kinarm-standard-tests>.
